# Aging and rejuvenation of active matter under topological constraints

**DOI:** 10.1038/s41598-017-05569-6

**Published:** 2017-07-18

**Authors:** Liesbeth M. C. Janssen, Andreas Kaiser, Hartmut Löwen

**Affiliations:** 1 Institute for Theoretical Physics II: Soft Matter, Heinrich-Heine University Düsseldorf, Universitätsstraße 1, 40225 Düsseldorf, Germany; 2 Materials Science Division, Argonne National Laboratory, 9700 South Cass Av, Illinois, 60439 USA; 3 Department of Applied Physics, Eindhoven University of Technology, P.O. Box 513, 5600MB, Eindhoven, The Netherlands

## Abstract

The coupling of active, self-motile particles to topological constraints can give rise to novel non-equilibrium dynamical patterns that lack any passive counterpart. Here we study the behavior of self-propelled rods confined to a compact spherical manifold by means of Brownian dynamics simulations. We establish the state diagram and find that short active rods at sufficiently high density exhibit a glass transition toward a disordered state characterized by persistent self-spinning motion. By periodically melting and revitrifying the spherical spinning glass, we observe clear signatures of time-dependent aging and rejuvenation physics. We quantify the crucial role of activity in these non-equilibrium processes, and rationalize the aging dynamics in terms of an absorbing-state transition toward a more stable active glassy state. Our results demonstrate both how concepts of passive glass phenomenology can carry over into the realm of active matter, and how topology can enrich the collective spatiotemporal dynamics in inherently non-equilibrium systems.

## Introduction

Active systems are composed of particles that can convert chemical, magnetic, or radiation energy into autonomous motion, rendering them intrinsically far from equilibrium^1–4^. Examples of living active matter are found on many length scales, from microscopic motile bacteria to macroscopic flocks of birds, and also numerous synthetic active materials have recently become available^5^. The spatiotemporal dynamics exhibited by such systems range from swarming and giant number fluctuations^6, 7^ to low-Reynolds-number turbulence^8–11^ and motility-induced phase separation^12–15^, illustrating the rich collective behavior that emerges from the non-equilibrium energy dissipation and active self-motility at the single-particle level.

It was recently found that sufficiently dense assemblies of active matter can also exhibit hallmarks of glassy dynamics^16–28^, including slow relaxation, dynamic heterogeneity, and ultimate kinetic arrest–akin to the behavior observed in non-active supercooled liquids and dense colloidal suspensions^29^. For passive systems, the process of glass formation has been widely studied over the last few decades, resulting in multiple compelling theoretical scenarios for the conventional glass transition^29–31^. However, the extent to which the phenomenology of passive glass-formers differs from that of non-equilibium dense active matter remains a topic of scientific debate. For example, while initial simulation studies suggested that adding activity generally pushes the glass transition to higher densities and lower temperatures^18, 19^, more recent work argues that active self-motility can both increase and decrease a system’s glassiness^21, 22^. This indicates that activity has a more intricate effect on the dynamics than merely shifting the effective density or temperature. The question whether time-dependent out-of-equilibrium glassy phenomena such as aging and rejuvenation may also occur in active matter has thus far remained unexplored. These latter processes are generally understood in terms of an energy-landscape picture, whereby aging and rejuvenation correspond to relaxation toward deeper and shallower energy minima, respectively^32^. However, owing to the non-Hamiltonian nature of particle activity, the potential (or free) energy is generally not a useful metric for active matter, and hence it remains unclear if and how aging and rejuvenation might be manifested in an active glass.

A different avenue of research concerns the effects of geometric^33–38^ and topological^39–46^ constraints on active matter. For passive soft matter systems, it is well established that confining a system to a curved surface can both frustrate and promote long-range orientational order^47–49^, induce complex topological-defect structures^50–53^, and affect a system’s glass-forming properties^54^. In a biological context, surface curvature is known to play a role in collective cell migration during e.g. embryonic development^55^ and the growth of the corneal epithelium^56^. For active soft-matter systems, however, only a limited number of experimental and theoretical studies has addressed the role of curvature and topology. Explicitly, recent experimental work has focused on active nematic microtubuli confined to a deformable droplet interface^39^, and subsequent theoretical^40–42^ and simulation^43–46^ studies have explored the dynamics of nematic and polar active particles under a spherical or ellipsoidal constraint. These developments point toward a rich array of topological-defect patterns and curvature-driven dynamics in the presence of strong aligning interactions between the particles. It remains unclear, however, how active particles with weak alignment interactions behave under topological constraints, and how disordered glass-like dynamics may possibly emerge under such conditions.

Here we seek to unite these independent lines of research and present a systematic study of the interplay between topology, particle activity, and effective particle alignment interactions. Specifically, we perform Brownian dynamics simulations of repulsive, self-propelled polar rods confined to a compact spherical manifold, and explore the emergent collective dynamics for different packing densities and particle aspect ratios. We find that particularly the high-density regimes are influenced by the confining topology, and for sufficiently dense short rods, we observe a novel glass transition toward a solid-like disordered state in which all particles undergo collective rotation. Remarkably, upon repeated melting and vitrification of this self-spinning glass phase, we also find evidence of aging and rejuvenation dynamics, which we clarify in terms of an absorbing-state formalism and a stability-landscape picture. Overall, our results exemplify both the novel spatiotemporal dynamics that may emerge from coupling activity to topology, and the surprising analogies between active matter that is intrinsically out-of-equilibrium, and passive glassy matter that is collectively out-of-equilibrium. Our findings might be tested in experiments on e.g. dense suspensions of bacterial or synthetic active particles confined to a spherical droplet or hydrogel interface.

## Results

### State diagram

**Figure 1:**
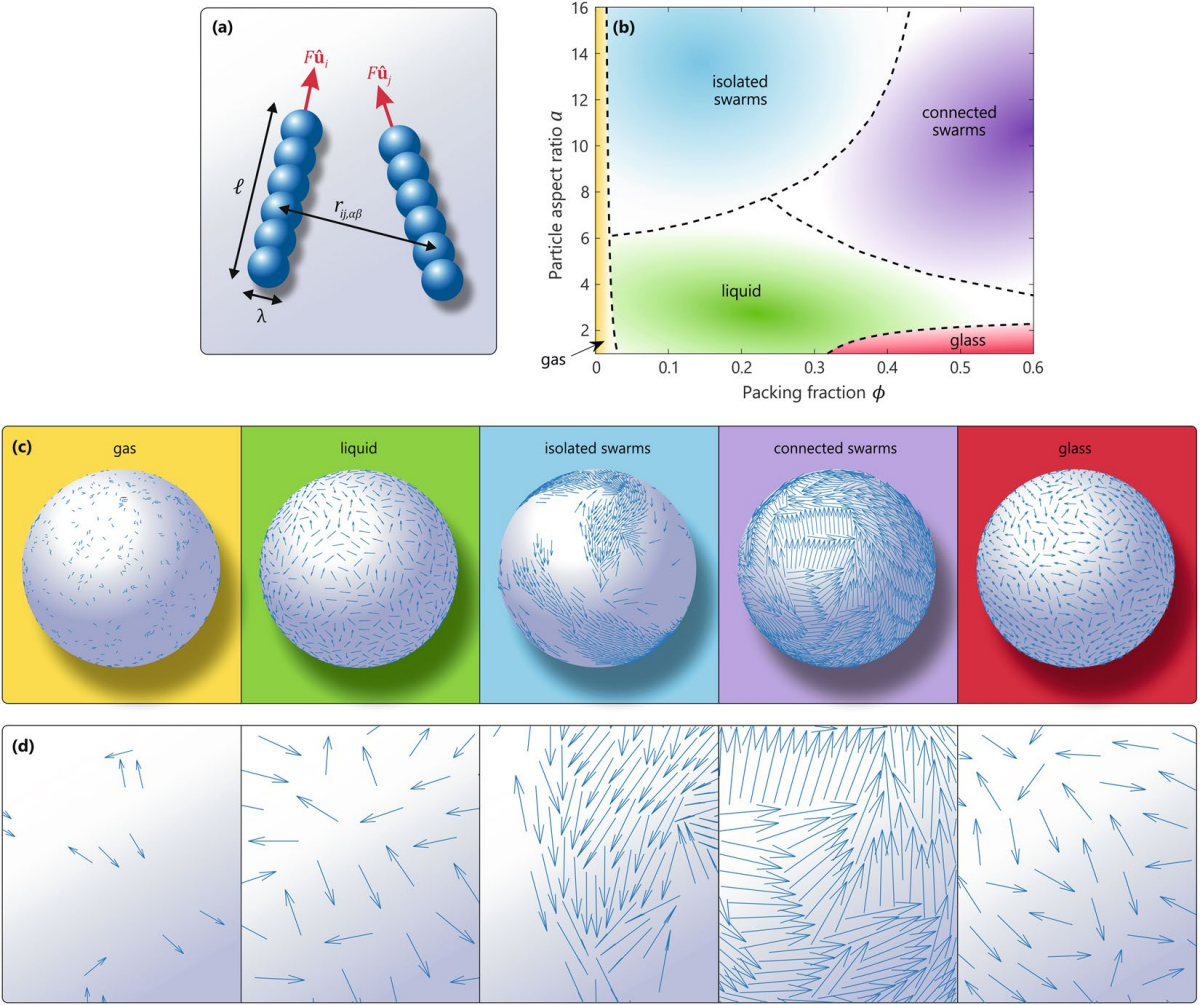
Non-equilibrium state diagram for active rods on a sphere. (**a**) Schematic representation of our active-rod model system. (**b**) State diagram for *N* = 800 active particles on a spherical surface as a function of the particle aspect ratio $a=\ell /\lambda$ and packing fraction *ϕ*. The different phases were identified by visual inspection of each indivual trajectory. Dashed lines indicate approximate boundaries between phases and serve as a guide to the eye. The evaluated state points are indicated in the Supplementary Information. (**c**) Typical snapshots of the different phases indicated in the state diagram: gas (*a* = 10, *ϕ* = 0.01), liquid (*a* = 4, *ϕ* = 0.2), isolated swarms (*a* = 16, *ϕ* = 0.1), connected swarms (*a* = 10, *ϕ* = 0.6), and glass (*a* = 2, *ϕ* = 0.5). (**d**) Corresponding close-ups of the snapshots. Every blue arrow represents a single particle with orientation vector ${\hat{{\bf{u}}}}_{i}$.

We first explore the full non-equilibrium state diagram of self-propelled rods on a sphere as a function of the packing fraction and particle aspect ratio. Our system is based on a suitable minimal model system for bacterial microswimmers in Euclidean space^8, 57^, which is illustrated in Fig. 1(a) and discussed in detail in the Methods section. Briefly, we consider *N* rigid, self-propelled rods of length $\ell$ that move with a constant self-propulsion force *F* directed along the main rod axis $\hat{{\bf{u}}}$. Each rod consists of *n* spherical segments that interact with the segments of any other rod through a steeply repulsive Yukawa potential, preventing particles to overlap. The screening length *λ* of the Yukawa interaction defines the effective width of the rods and serves as our unit of length. We perform a series of overdamped Brownian dynamics simulations as a function of the particle aspect ratio $a=\ell /\lambda$ and effective packing fraction $\varphi =N\ell \lambda /\mathrm{(4}\pi {R}^{2})$, where *R* denotes the radius of the confining sphere. Throughout the simulations, the rods are constrained to lie tangent to the surface of the confining spherical manifold, with each rod’s center-of-mass position **r**_*i*_ connected to the sphere. For simplicity we ignore hydrodynamic interactions and thermal noise, thus allowing us to focus on a minimal model system that captures the interplay between the particles’ geometry, packing density, and topology of the confining sphere. Finally, considering the inherent finize size of a spherical surface, which implicitly prevents the existence of a thermodynamic limit, we restrict ourselves to the behavior of small systems of typically 400–800 rods.

Figure 1(b) shows the state diagram of our spherically constrained active-rod system as a function of the rod aspect ratio *a* and packing fraction *ϕ*, calculated for a system of *N* = 800 rods. Snapshots of the corresponding phases are shown in Fig. 1(c,d), and the time-dependent dynamics can be seen in Supplementary Movies S1 to S5. With the exception of extremely dilute packings $\varphi \mathop{ < }\limits_{ \tilde {}}0.01$–in which case an active-gas phase forms–we can identify a marked dependence on rod length in the dynamical behavior. For *large* particle aspect ratios, we find that the rods tend to align and spontaneously form domains of local polar order. This alignment effect is well established for active repulsive rods in 2D Euclidean space, and here we find that it also applies in curved space. The observed alignment is the result of pair collisions: when two active rods collide, the resulting torques and steric forces cause the rods to orient in the same direction and move close to each other–even though no attractive forces exist between the particles^58^. At low packing fractions, this leads to a distinct *swarming* phase in which the rods group together in isolated flocks and exhibit giant density fluctuations, completely analogous to swarming in Euclidean space^57^. For higher packing fractions, however, the rods experience a packing constraint and become affected by the presence of the confining topology: the different swarms become connected and form a giant, dynamic “multi-domain swarm” that ultimately spans the entire sphere. As in the lower-density swarming phase, each of these domains is composed of locally oriented rods with polar and/or smectic order. At sufficiently high densities, transient topological defects can be identified at the boundaries between the different domains, and the dynamics becomes a rich pattern of mobile defects and transient counter flows.

We remark that in the limit of an infinite sphere radius (or infinite particle number *N*), our state diagram should extrapolate to that for a flat 2D surface. The latter contains a distinct turbulent and laning phase for long rods at high density^57^, while in our current work we can identify only a “connected swarms” phase. The fact that we do not observe a well-developed turbulent phase here is likely due to the relatively small number of particles used, preventing the formation of a coarse-grained vorticity field. However, the fact that we do not observe a clear laning phase is inherently due to the confining topology: at least for small system sizes of *N* = 800 rods, we have verified that a flat 2D surface with periodic boundary conditions quickly gives rise to distinct laning, while on the sphere such a phase is never stable. Thus, if the system size is sufficiently small to “feel” the presence of the confining spherical topology, the 2D global laning phase is destabilized and converted into the dynamic “connected swarms” phase that exhibits only local and transient laning-like behavior.

The emergent dynamics becomes dramatically different when reducing the particle aspect ratio *a*. Short rods experience only a small torque during a pair collision, causing the alignment effect to eventually vanish and consequently giving rise to strongly disordered dynamics. Indeed, for short rods at low packing fractions, we observe an active-liquid phase in which the particles move incoherently and exhibit no strong cooperative motion. Note that in this state, in contrast to the long-rod case, the particles are all oriented randomly and are spread homogeneously across the surface of the sphere.

Intriguingly, we find that at sufficiently dense packings, systems with *a* < 2.5 undergo a marked transition into a kinetically arrested state, as depicted in Fig. 1 and Supplementary Movie S5. In this non-ergodic phase, which we term a *self*-*spinning glass*, the relative positions and orientations of all particles are frozen in a disordered configuration, but the system as a whole undergoes a *collective* rotation about a fixed arbitrary axis with constant angular velocity. The source of this self-sustained spinning dynamics lies in the activity: every particle in the glassy state exerts a constant self-propulsion force *F* in a (quasi-)random direction, giving rise to a net (random) force that in general will be small but nonzero. This, in turn, produces a finite torque that drives the collective rotation. Note that such a spinning motion is a consequence of the unique topology of the sphere and would be unattainable on a flat 2D plane–the latter permitting only collective translational motion, as indeed also found in ref. 57. The active spinning behavior is reminiscent of the rotational dynamics found in multicellular spherical Volvox colonies^59^, but differs in the sense that the glass phase lacks any orchestrated mechanism to direct the individual particles’ activity.

### Dynamics of the self-spinning active glass

**Figure 2:**
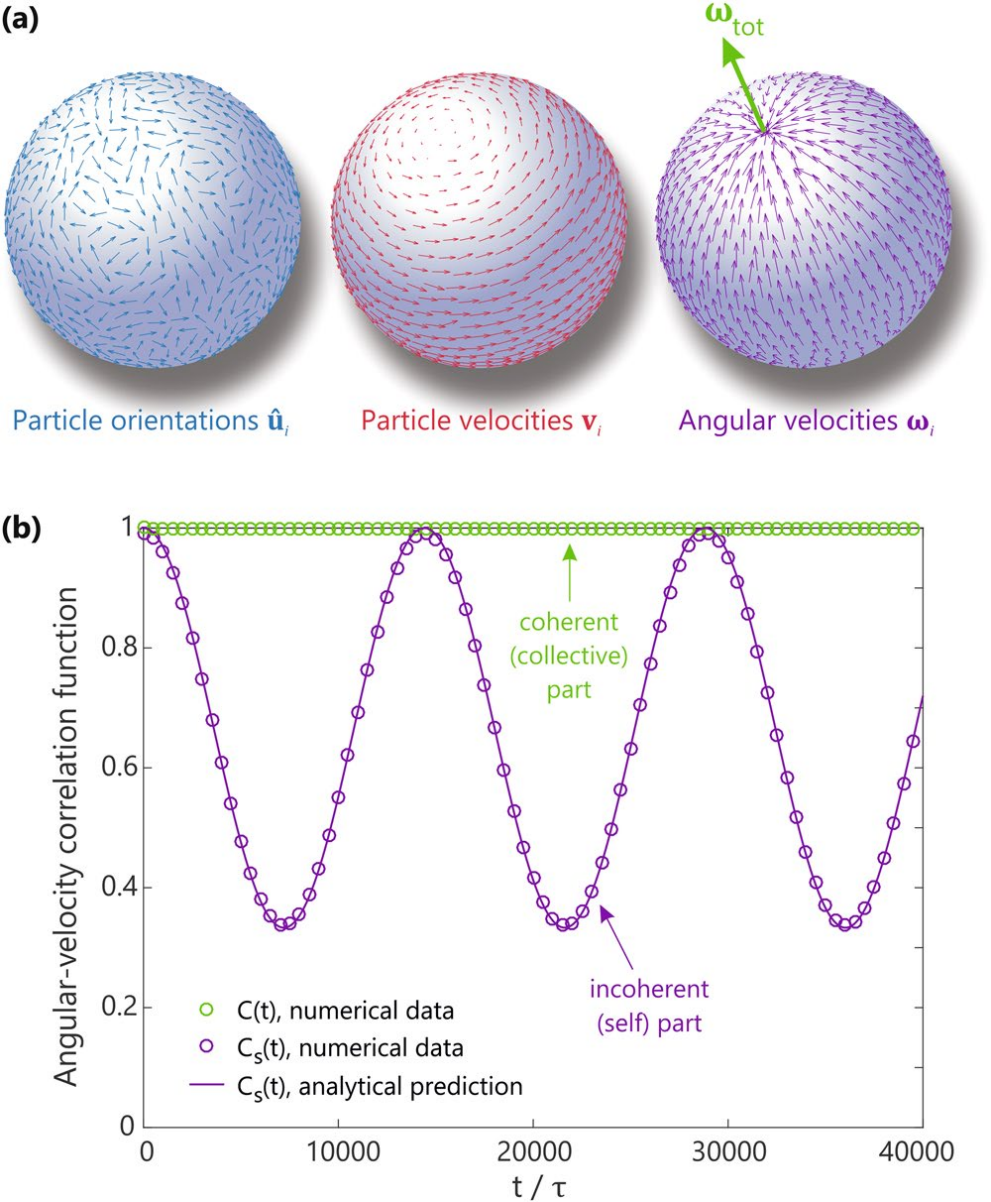
Angular velocities in the self-spinning glass phase. (**a**) Snapshots of the particle orientations ${\hat{{\bf{u}}}}_{i}$ (blue arrows), instantaneous velocities **v**_*i*_ (red arrows), and normalized angular velocities ${\hat{{\boldsymbol{\omega }}}}_{i}$ (purple arrows) for an arbitrary glassy configuration of *N* = 800 particles with aspect ratio *a* = 2 at packing fraction *ϕ* = 0.5. (**b**) Corresponding time correlation functions *C*_*s*_(*t*) and *C*(*t*), probing the self- and collective parts of the angular-velocity autocorrelation, respectively. The solid purple line indicates the analytical prediction of *C*_*s*_(*t*) for a rotation period of *T* = 14410*τ*.

In order to characterize the self-spinning motion, let us focus on the angular velocity field in the glass phase. Figure 2(a) depicts a snapshot of the typical particle orientations ${\hat{{\bf{u}}}}_{i}$, instantaneous velocities **v**_*i*_, and corresponding angular velocity field for a glass of *N* = 800 particles with aspect ratio *a* = 2 and packing fraction *ϕ* = 0.5, where the normalized angular velocity for each particle *i* is defined as ${\hat{{\boldsymbol{\omega }}}}_{i}=({{\bf{r}}}_{i}\times {{\bf{v}}}_{i})/|{{\bf{r}}}_{i}||{{\bf{v}}}_{i}|$. The total angular velocity, defined as ${\omega }_{{\rm{tot}}}={\sum }_{i}\,{{\bf{r}}}_{i}\times {{\bf{v}}}_{i}$, is a vector pointing in the direction of the rotation axis, whose norm $|{{\boldsymbol{\omega }}}_{{\rm{tot}}}|$ quantifies the global angular speed of rotation. The time-dependent dynamics of the spinning motion is now conveniently captured in the autocorrelation function of the angular velocity. To this end, we make a distinction between the incoherent or self-part of the correlation function

1 $${C}_{s}(t)=\frac{{\sum }_{i}\langle {\hat{{\boldsymbol{\omega }}}}_{i}\mathrm{(0)}\cdot {\hat{{\boldsymbol{\omega }}}}_{i}(t)\rangle }{{\sum }_{i}\langle {\hat{{\boldsymbol{\omega }}}}_{i}\mathrm{(0)}\cdot {\hat{{\boldsymbol{\omega }}}}_{i}\mathrm{(0)}\rangle },$$

and the coherent or collective part

2 $$C(t)=\frac{\langle {{\boldsymbol{\omega }}}_{{\rm{tot}}}\mathrm{(0)}\cdot {{\boldsymbol{\omega }}}_{{\rm{tot}}}(t)\rangle }{\langle {{\boldsymbol{\omega }}}_{{\rm{tot}}}\mathrm{(0)}\cdot {{\boldsymbol{\omega }}}_{{\rm{tot}}}\mathrm{(0)}\rangle },$$

where *t* denotes time and the brackets are appropriate statistical averages. As discussed below, these two functions offer valuable and complementary insight into the time-dependent dynamics of the system.

Figure 2(b) shows the time-dependent behavior of both correlation functions, calculated for the glassy state depicted in Fig. 2(a), where the statistical average is taken over different time origins. The incoherent function *C*_*s*_(*t*) clearly reveals a steady non-vanishing rotational motion, with the period of rotation determined by the net angular speed $|{{\boldsymbol{\omega }}}_{{\rm{tot}}}|$. We point out that this oscillation period is essentially arbitrary; a different random starting configuration will equilibrate to a different disordered state, giving rise to a different net angular velocity. Indeed, we have performed tests for 1000 different initial conditions, and found that the Cartesian components of ${{\boldsymbol{\omega }}}_{{\rm{tot}}}$ are normally distributed around zero, consistent with the Central Limit Theorem. Also note that *C*_*s*_(*t*) oscillates between the values of 1 and 1/3, which is a consequence of the geometry of the spherical surface: in the stable glass phase, the angular velocity of a particle at the pole will anti-correlate with itself after half a period of rotation, while a particle at the equator will have a constant angular velocity. The total particle average as a function of time, assuming homogeneous coverage of the sphere, is then ${C}_{s}(t/T)={\int }_{0}^{1}\,dv[{(2v-1)}^{2}\,\cos (2\pi t/T)-{(2v-1)}^{2}+1]=\frac{1}{3}[\,\cos (2\pi t/T)+2]$, where *T* is the total period of rotation. As can be seen in Fig. 2(b), this analytical result is in perfect agreement with our numerical results. For the *coherent* correlator *C*(*t*), however, the curvature and topology of the confining geometry do not play any role, since the total angular velocity ${{\boldsymbol{\omega }}}_{{\rm{tot}}}$ is constant in the glassy state. Hence the normalized collective autocorrelation function will always be 1 in this case. Overall, these result confirm that the self-spinning glass state is a highly robust phase that continues to spin indefinitely in an arbitrary but fixed direction.

### Melting and revitrification dynamics

**Figure 3:**
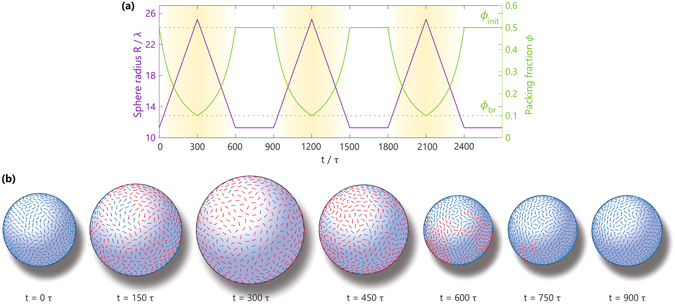
Breathing protocol to induce melting and revitrification of the glass phase. (**a**) Protocol for three consecutive breathing cycles of periodic inflation and deflation of the sphere. A single breathing cycle consists of three stages: first we dilute the system from a packing fraction *ϕ*_init_ to *ϕ*_br_ by linearly increasing the sphere radius *R* in 30 steps, allowing the system to briefly equilibrate at every new *R*-value for a duration of 10*τ*. We subsequently re-densify the system to the original packing fraction *ϕ*_init_ by a stepwise linear decrease in *R* over a time period of 30 × 10*τ*, and finally we allow the system to re-equilibrate at *ϕ* = *ϕ*_init_ during a time interval of 300*τ*. In this example we have *ϕ*_init_ = 0.5 and *ϕ*_br_ = 0.1, as indicated by the green dashed lines. (**b**) Particle snapshots for a single breathing cycle with *ϕ*_init_ = 0.5 and *ϕ*_br_ = 0.2 (also see Supplementary Movies S6 and S7). Blue arrows indicate immobile particles whose centers of mass have moved less than a distance 0.2*λ* within a time frame of 20*τ*, while red arrows indicate mobile particles that have moved more that 0.2*λ* during the same time interval. Notice that at low densities the system melts and almost all particles undergo large displacements, while at high densities the system locks into a glassy phase in which, aside from the overall spinning motion, no particles rearrange.

We now turn our attention to the dynamics that emerges upon melting and revitrification of the spinning glass phase. The relevant control parameter that drives the glass transition in our system is the packing fraction, and hence the active glass can be melted by increasing the size of the confining sphere while keeping the particle number *N* constant. The revitrification process may subsequently be induced by compressing the sphere to a smaller radius, thus effectively increasing the packing fraction again. In order to systematically study the effect of fluctuations in the packing fraction, we introduce a “breathing protocol” whereby the sphere is periodically inflated and deflated to a certain upper and lower radius, respectively, allowing us to switch repeatedly between the ergodic active-fluid phase and the dense glassy state. Figure 3(a) illustrates the protocol for three consecutive cycles that switch between packing fractions *ϕ* = 0.5 and *ϕ* = 0.1, and Fig. 3(b) shows typical snapshots of particle configurations during one cycle (also see Supplementary Movies S6 and S7). In general, a single breathing cycle starts at a packing fraction *ϕ*_init_, and is then diluted to *ϕ*_br_ < *ϕ*_init_ by linearly increasing the sphere radius *R* in 30 steps. The system is subsequently re-densified toward *ϕ*_init_ by linearly decreasing *R* in 30 steps, followed by a final stage in which we keep the packing fraction constant at *ϕ* = *ϕ*_init_ (see Methods). We note that this protocol is somewhat reminiscent of other periodic driving schemes that are commonly applied to passive glasses, such as oscillatory shearing^60^ and thermal cycling^61, 62^. However, our breathing protocol amounts to a periodic change in density, while shearing and thermal cycling keep the density constant.

**Figure 4:**
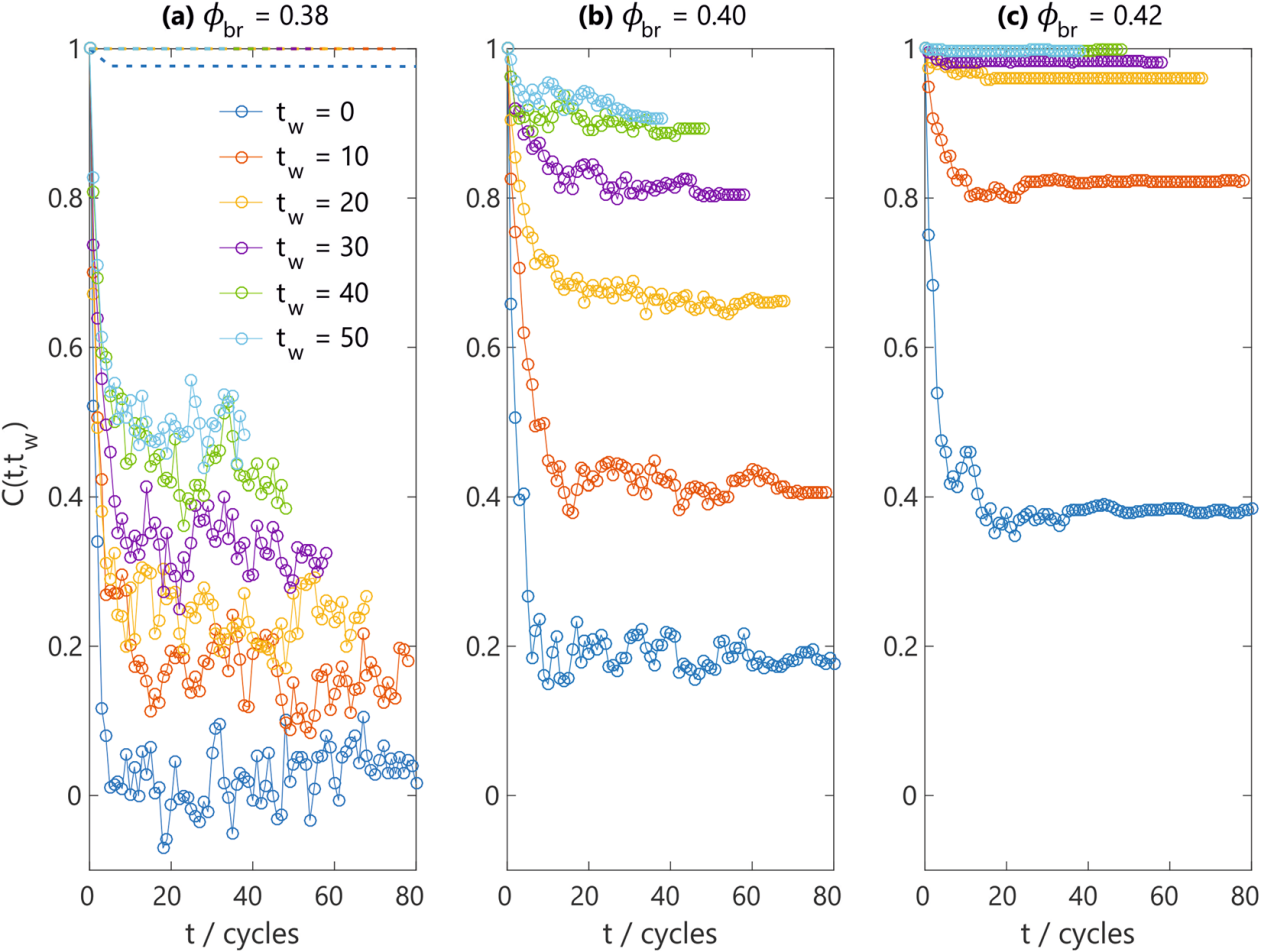
Aging dynamics in the active glass phase upon melting and re-vitrification. Time correlation function of the total angular velocity, *C*(*t*, *t*_*w*_), for different waiting times *t*_*w*_ and different breathing amplitudes *ϕ*_br_: (**a**) *ϕ*_br_ = 0.38, (**b**) *ϕ*_br_ = 0.40, and (**c**) *ϕ*_br_ = 0.42, all starting from the active glass phase at *ϕ*_init_ = 0.5. The data were collected for *N* = 400 particles with *a* = 2, averaged over 100 independent equilibrated starting configurations. As a reference, we also show the results for a *passive* system without any self-propulsion, plotted as dashed lines in panel (a) for *ϕ*_br_ = 0.38.

Let us investigate the dynamics of the system as a function of the number of applied breathing cycles. To this end, we introduce a time-dependent angular-velocity correlation function

3 $$C(t,{t}_{w})=\frac{\langle {\hat{{\boldsymbol{\omega }}}}_{{\rm{tot}}}({t}_{w})\cdot {\hat{{\boldsymbol{\omega }}}}_{{\rm{tot}}}(t+{t}_{w})\rangle }{\langle {\hat{{\boldsymbol{\omega }}}}_{{\rm{tot}}}({t}_{w})\cdot {\hat{{\boldsymbol{\omega }}}}_{{\rm{tot}}}({t}_{w})\rangle },$$

which now depends explicitly on the waiting time *t*_*w*_. Here, ${\hat{{\boldsymbol{\omega }}}}_{{\rm{tot}}}$ is the normalized total angular velocity and the brackets denote an average over different independent configurations (also see Methods). For convenience, we will quantify *t*_*w*_ in units of the applied number of cycles, with each cycle representing a time span of 900*τ*. Interestingly, we find a distinctly different behavior of *C*(*t*, *t*_*w*_) depending on the magnitude of the fluctuations in the packing fraction. Figure 4 compares the dynamics in a system of *N* = 400 particles (*a* = 2) for different breathing amplitudes of *ϕ*_br_ = 0.38, 0.40, and 0.42, all starting from a glassy phase at *ϕ*_init_ = 0.5. For the largest expansion amplitude considered, *ϕ*_br_ = 0.38, it can be seen that *C*(*t*, *t*_*w*_) rapidly decays to zero if *t*_*w*_ = 0 (i.e., before applying any expansion-compression cycle), but builds up an increasingly large nonzero long-time limit as the number of applied cycles increases. This signifies that the total angular velocity in the glassy state becomes increasingly more correlated to that of all future revitrified configurations. Applying a breathing protocol with a slightly smaller change in density, e.g. *ϕ*_br_ = 0.40, enhances this effect. In view of this marked dependence on waiting time, which we observe both in *N* = 400 and *N* = 800 systems under moderately small breathing amplitudes, we assert that our system is *aging*.

It is important to realize that the particles’ inherent activity is a crucial ingredient for the aging process; an equilibrated *passive* system without self-propulsion will–in the absence of noise–remain in the same configuration indefinitely, regardless of breathing amplitude and waiting time *t*_*w*_. Indeed, as shown for comparison for *ϕ*_br_ = 0.38 in Fig. 4(a), a strictly passive reference system rapidly yields a constant correlation value *C*(*t*, *t*_*w*_) = 1, exhibiting only a marginal decorrelation effect at very short times (also see Supplementary Movie S8). This stark contrast between the active and passive time-dependent dynamics confirms that the observed aging phenomenon is indeed activity-induced.

The degree of aging is, however, sensitive to the relative amplitude of the breathing motion. Under the mildest breathing protocol considered here, *ϕ*_br_ = 0.42, the correlator *C*(*t*, *t*_*w*_) already attains its maximal value of 1 after 40 full cycles, and hence no more aging dynamics can be observed for all longer waiting times *t*_*w*_ > 40. In the extreme limit of *ϕ*_br_ = *ϕ*_init_, the system remains frozen in its original spinning-glass configuration for all times *t* and *t*_*w*_, thus causing the aging effect to vanish completely. As a final case, let us consider the opposite limit of *ϕ*_br_ → 0, which allows the system to melt into a dilute fluid phase during every cycle (also see Supplementary Movie S6). For such large-amplitude breathing, *C*(*t*, *t*_*w*_) will rapidly decay to zero, independent of the number of cycles *t*_*w*_. That is, the re-solidification stage from the melt at *ϕ*_br_ to *ϕ*_init_ will always yield a new glassy configuration that is completely uncorrelated to the orientation of ${{\boldsymbol{\omega }}}_{{\rm{tot}}}$ at the beginning of the cycle. In analogy to the phenomenology in oscillatory-sheared passive systems^63^, we will refer to such a process as *rejuvenation*: each full breathing cycle will wash away any possible memory of the original glassy state and produce a new self-spinning glass with an entirely new ${{\boldsymbol{\omega }}}_{{\rm{tot}}}$.

### Activity-induced aging mechanism

We now seek to gain more insight into the physical mechanism that underlies the observed activity-induced aging dynamics. Upon inspection of the particle trajectories for *ϕ*_init_ = 0.5 and ${\varphi }_{{\rm{br}}}\approx 0.4$, we find that the system generally does not melt completely, but rather exhibits a limited amount of cooperative particle rearrangements–strongly reminiscent of the dynamically heterogeneous dynamics observed in normal glass-forming liquids. As the aging process further evolves, the average number of rearranging particles tends to decrease and ultimately the system locks into a new configuration in which all relative particle motion has ceased. That is, the system has seemingly reached a glassy state that is sufficiently stable to sustain a breathing amplitude of *ϕ*_br_, and consequently remains in this stable state indefinitely (see Supplementary Movie S7).

**Figure 5:**
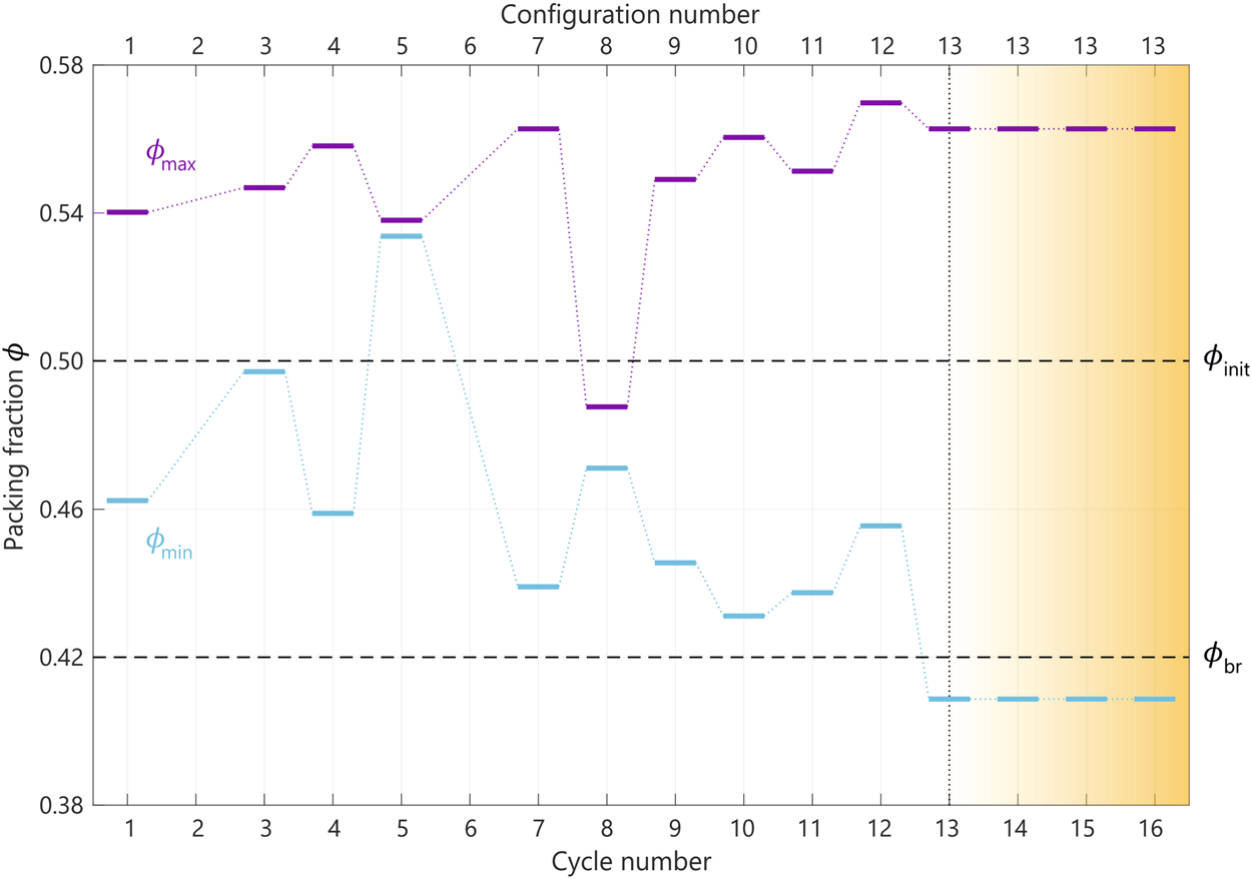
Stability of the active-particle configurations formed during aging. Stability analysis of the configurations obtained from a single trajectory for *N* = 800 active particles with self-propulsion strength *F* = 1, undergoing 20 consecutive breathing cycles for *ϕ*_init_ = 0.5 and *ϕ*_*br*_ = 0.42. The stability was measured for the final configuration after every cycle. The purple lines indicate the maximum packing fraction, *ϕ*_max_, at which a configuration is still stable; for *ϕ* > *ϕ*_max_ the system will undergoing particle rearrangements to avoid unphysical particle overlaps. The blue lines indicate the minimum packing fraction, *ϕ*_min_, at which a configuration is still stable; for *ϕ* < *ϕ*_min_ the system will melt into an active fluid phase. Note that after 13 cycles, the system locks into a configuration that is stable at all *ϕ*_br_ < *ϕ* < *ϕ*_init_, and hence remains in this configuration for all remaining cycles (shaded yellow region).

In order to quantify the emergent stability of the particle configurations during aging, we use the total number of rearranging particles *N*_*r*_ as a metric and determine at which packing fraction a given configuration will become unstable such that *N*_*r*_ > 0. Here we define particle rearrangement using a Lindemann-like criterion for melting, as described in the Methods section. Figure 5 shows the results of our stability analysis for a single aging trajectory of *N* = 800 active particles undergoing 16 consecutive breathing cycles between *ϕ*_init_ = 0.5 and *ϕ*_br_ = 0.42. The stability was measured for every configuration at the end of a full cycle. Let us first point out two general observations with respect to Fig. 5: first of all, there need not exist any value of *ϕ* for which a given configuration is stable. Indeed, configuration numbers 2 and 6 in Fig. 5 are unstable for all possible packing fractions. Secondly, if there exists a range of *ϕ* values for which an active configuration is stable, the stability range will be bounded both from above and from below. Figure 5 shows these upper and lower packing fractions–denoted as *ϕ*_max_ and *ϕ*_min_, respectively–for all the remaining configurations of the trajectory. The reason for this boundedness is as follows: at high packing fractions *ϕ* > *ϕ*_max_, the particles are forced to rearrange in order to avoid unphysical overlaps due to the short-range repulsive interactions. For low packing fractions *ϕ* < *ϕ*_min_, the distance between particles becomes sufficiently large to facilitate quasi-ergodic particle motion, causing the system to ultimately melt into an active fluid phase. It is important to note that the latter lower bound does not exist for passive systems: particles with zero self-propulsion will become completely immobile (*N*_*r*_ = 0) in the limit of *ϕ* → 0, thus rendering them strictly stable in our definition. We will expand upon this point in the Discussion section.

Let us now return to the activity-induced aging phenomenon. Figure 5 reveals that the stability of the configurations formed during a single breathing trajectory does not monotonically increase with time; on the contrary, we observe an erratically varying pattern of stabilities, including intermittent states (after cycle 2 and 6) that are strictly unstable for all *ϕ*. After 13 cycles, however, the system reaches a configuration whose stability range spans the *entire* amplitude of the breathing motion, i.e., *ϕ*_max_ > *ϕ*_init_ and *ϕ*_min_ < *ϕ*_br_. Once this stable state is reached, the applied breathing protocol can no longer destabilize the configuration, consequently prohibiting the system to explore any other configurations in the remaining cycles. In close analogy to work on periodically driven systems^64^, we thus conclude that our system has undergone an irreversible, random self-organization process toward an “absorbing state” in which all particle fluctuations have vanished. It is this mechanism that underlies the observed aging: the system continues to explore many different configurations until it spontaneously reaches a stable state from which it can no longer escape.

We can define the absorbing state more generally as the firstly formed configuration with stability bounds *ϕ*_max_ ≥ *ϕ*_init_ and *ϕ*_min_ ≤ *ϕ*_br_; note that this state is principally one of infinitely many possible configurations. For moderately breathing amplitudes, such a stable state may always be reached provided that the waiting time is sufficiently long, as can be seen from Fig. 4. Conversely, for a strictly passive system with zero self-propulsion, *any* configuration in which the particles do not overlap too strongly can act as an absorbing state, and hence we observe no notable aging dynamics in the passive case.

**Figure 6:**
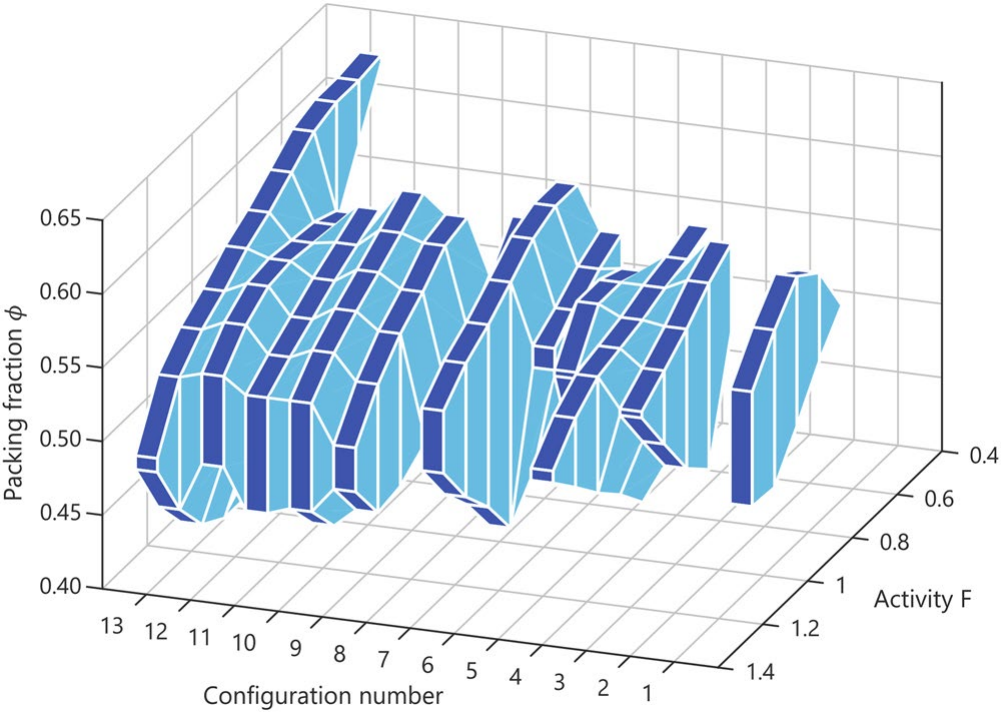
Stability of active configations as a function of self-propulsion strength. Stability analysis for the same configurations as in Fig. 5, but for different activities *F*. Dark-blue shaded areas enclose the regions of stability (*ϕ*_min_ ≤ *ϕ* ≤ *ϕ*_max_). Note that in this specific example, configuration number 2 remains unstable for all possible values of *F* > 0 considered; such a configuration can only be stabilized for *F* = 0 in the non-interacting gas limit *ϕ* → 0. Configuration number 6, which is unstable for *F* = 1, becomes stable for 0.6 ≤ *F* ≤ 0.9.

Finally, let us investigate how the stability of the formed configurations–and thus the nature of the absorbing state–is affected by the magnitude of the self-propulsion force *F*. As a proof-of-principle study, we have measured the stability of the 13 unique particle configurations considered in Fig. 5 for different values of *F*, thereby keeping the initial particle positions and orientations the same as for the *F* = 1 reference case (see Methods). The results are shown in Fig. 6. Clearly, the stability dependence for a given configuration on *F* is highly non-monotonic: the upper (*ϕ*_max_) and lower (*ϕ*_min_) stability bounds can both increase or decrease with increasing *F*, and also the total width of the stability range, i.e. *ϕ*_max_ − *ϕ*_min_, depends strongly on the exact configuration and value of *F*. In view of these results, we conclude that the set of possible absorbing states will generally be different for different values of the self-propulsion strength. This may also be rationalized by considering that the stability in our active glassy system arises from a delicate balance between the intrinsic self-propulsion and repulsive pair-interaction forces on the particles; changing the magnitude of the active forces will generally alter the force balance across the entire disordered network, giving rise to either reduced or enhanced local stability in the system. Consequently, the first absorbing state that a system finds is sensitively dependent on the exact value of *F*.

## Discussion

**Figure 7:**
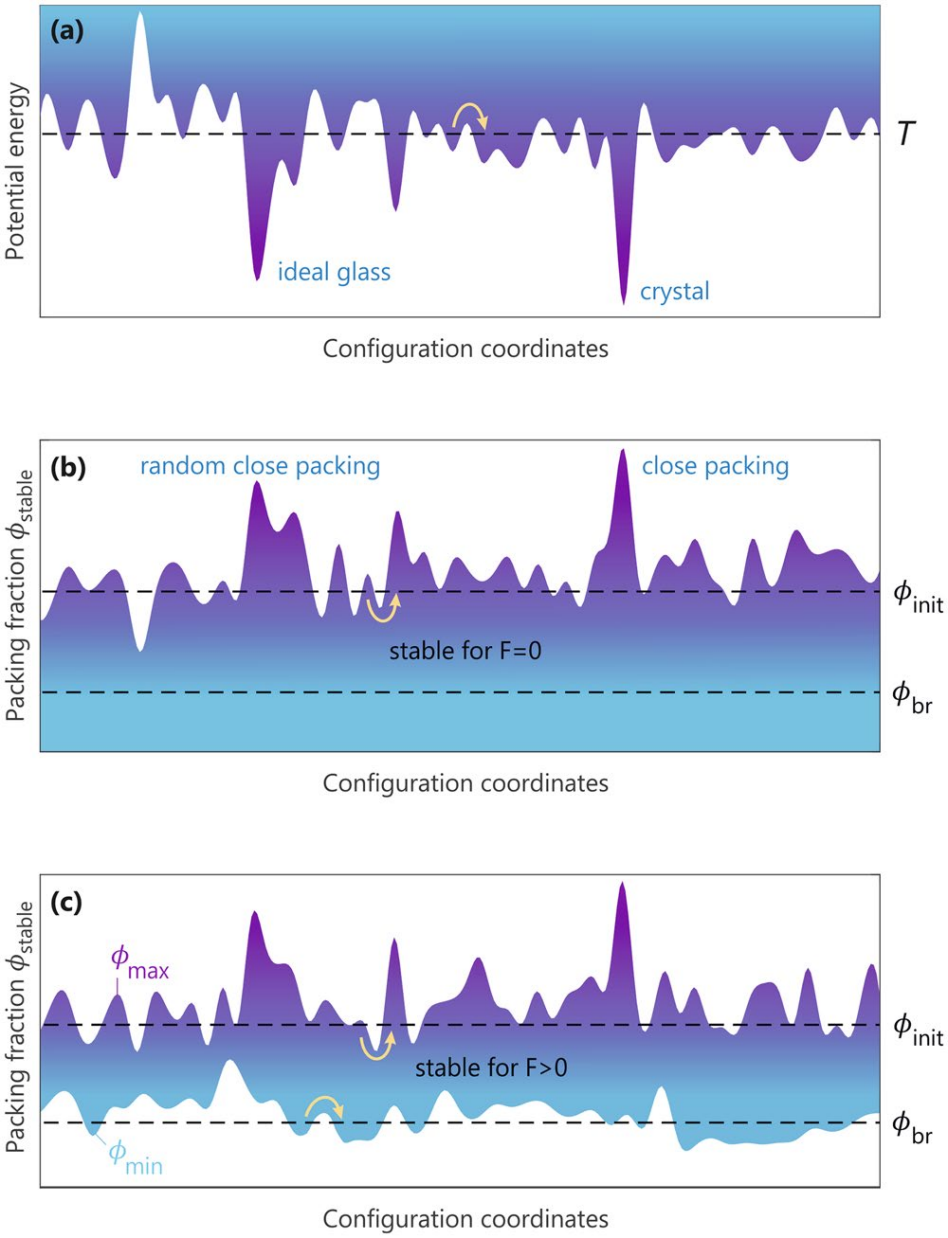
Schematic illustration of the landscape picture in glassy physics. The *x*-axis represents all configurational coordinates of an *N*-particle system. (**a**) The traditional potential-energy landscape of passive glass-forming systems, adapted from ref. 66, with a typical temperature *T* indicated by the dashed line. The global minimum of the energy is assumed to be the crystalline state, while the lowest energy state for a disordered configuration is the ideal glass. (**b**) Schematic stability landscape of passive (*F* = 0) glass-formers. The blue-shaded region marks the range of packing fractions for which the different configurations are stable, with stability defined here in terms of a Lindemann-like melting criterion. The global stability maximum is, by definition, the close-packing configuration, and for disordered systems the maximum corresponds to random close packing. (**c**) Schematic stability landscape of active (*F* > 0) glass-forming systems. Every active configuration will generally melt at sufficiently low density, and consequently the lower stability bound *ϕ*_min_ must be larger than zero. The dashed lines in panels (b,c) indicate the range of packing fractions, *ϕ*_br_ ≤ *ϕ* ≤ *ϕ*_init_, in which we prepare the system. The yellow arrows indicate typical barrier-crossing events to different parts of configuration space.

As discussed earlier, the observed aging dynamics occurs only for inherently active systems with a nonzero self-propulsion strength *F* > 0. Let us now compare this novel activity-induced aging mechanism with conventional aging in passive glass-forming systems. A key paradigm in the phenomenology of non-active glasses is the potential energy landscape^65–67^–a generally highly complex and rugged surface that describes the total potential energy of the system as a function of the 3*N*-dimensional configuration space [see Fig. 7(a)]. Within this landscape picture, aging and rejuvenation are understood as out-of-equilibrium processes whereby the system visits deeper or shallower local energy minima, respectively. The energy barriers separating these minima may be surmounted due to thermal fluctuations; if the system is prepared at a temperature *T*, the typical barrier height that can be crossed is on the order of *k*_B_ *T*, with *k*_B_ denoting the Boltzmann constant. This passive energy-landscape scenario is illustrated schematically in Fig. 7(a). Note that here the global energy minimum corresponds to the crystalline state, and the lowest minimum for a disordered configuration is referred to as the ideal glass state. For inherently active systems, however, the total potential energy is not neccessarily a useful metric, since the self-propulsion of the particles requires a constant (implicit) source of energy. Indeed, we also find that the total potential energy of our active system is generally not minimized during aging, implying that the aging process in passive glasses is not equivalent to our active-matter case.

Instead, we argue that the observed active aging and rejuvenation dynamics can be associated with a rugged “stability landscape” that quantifies the mechanical stability of all possible particle configurations. Such a landscape is essentially the 3*N*-dimensional generalization of Fig. 5 discussed in the previous section, where we have defined stability in terms of a Lindemann-like melting criterion. Let us first consider the passive version of this landscape. Since a configuration with *F* = 0 (in the absence of noise) will always be stable such that *N*_*r*_ = 0 in the dilute limit *ϕ* → 0, we have a rigorous minimum stability bound *ϕ*_min_ = 0 and a maximum bound *ϕ*_max_ that depends on the exact configuration. Figure 7(b) shows a schematic representation of this passive scenario. Note that the global maximum of *ϕ*_max_ is, by definition, the close-packing configuration, and for *disordered* systems the maximum attainable value of *ϕ*_max_ is at random close-packing.

For an active system with *F* > 0, however, the shape and properties of the stability landscape become decidedly different: first of all, all active configurations must melt at sufficiently low density, so that *ϕ*_min_ > 0. Moreover, activity may both enhance and reduce the stability of a given configuration, and hence the positions of local minima and maxima will generally shift with varying *F*. It must also be noted that certain configurations can become strictly unstable for all *F* > 0, as we found for e.g. configuration number 2 in Figs 5 and 6, giving rise to open voids in the stability landscape; however, in analogy to the inherent-structure formalism for passive glasses^66–68^, we assume that any instantaneous configuration can be quenched to a nearby state with a finite stability range, and the landscape of such inherent structures will be devoid of voids. With this active-stability picture in mind, as illustrated in Fig. 7(c), we can interpret the aging process as follows: during a dynamics simulation with periodic breathing, the system will explore different regions of the landscape until it reaches an absorbing state that is characterized by *ϕ*_max_ ≥ *ϕ*_init_ and *ϕ*_min_ ≤ *ϕ*_br_. Rejuvenation can occur by subsequently increasing the amplitude of breathing to *ϕ*_br_ < *ϕ*_min_, inducing a (partial) melt to allow the system to explore new regions of configuration space, until eventually a new absorbing state is reached with higher stability. We emphasize that this aging and rejuvenation analysis should apply generally to *any* active glassy system, regardless of the system size and topology, and is thus not limited to the spherical active-rod model of the present study.

As a final point, let us elaborate on the role of noise in the observed active aging dynamics. In the noise-free case, the aging process ceases as soon as the active system reaches an absorbing state; however, if noise is added by introducing fluctuations Δ*ϕ* in the breathing amplitude, the system might be able to escape from an absorbing state and cross local barriers on the stability landscape whose heights are on the order of Δ*ϕ*. Such fluctuations would essentially play the role of thermal fluctuations in the passive case, and would cause the active aging process to continue indefinitely. Indeed, just as a passive thermal glass will age by visiting increasingly deeper energy minima, our active glass is expected to reach increasingly more stable states as it ages under a weakly fluctuating breathing motion. This barrier-crossing process is illustrated schematically in Fig. 7; note that for active glasses a stability barrier may exist both in *ϕ*_max_ and *ϕ*_min_. In addition to this source of fluctuations, we may also consider *thermal* noise in our system, which can give rise to stochastic fluctuations in the particles’ centers of mass and orientations. In such a case, we expect every absorbing state to be replaced by a *basin* of absorbing states, analogous to the passive potential-energy landscape picture where basins emerge as deep energy minima that are separated by relatively small barriers. Importantly, however, within the current stability-landscape picture, thermal noise can also act as a proxy for activity: a passive particle system will, in the presence of thermal fluctuations, melt at sufficiently low densities. Hence, the lower stability bound *ϕ*_min_ will always become greater than zero, akin to the noise-free case of Fig. 7(c) for active systems. Finally, we note that the existence of noise may also provide opportunities for encoding memory into an active system, similar to recent studies on passive model glass-formers under oscillatory shear^69^.

In conclusion, we have explored the emergent dynamics in an active-matter system constrained to a spherical manifold. In the absence of strong aligning forces, we find that active particles at sufficiently high density can undergo a glass transition towards a non-ergodic state that is characterized by persistent collective spinning motion. Upon repeated melting and revitrification of such a self-spinning glass, we observe signatures of non-equilibrium aging and rejuvenation that occur solely for strictly active systems. We rationalize the activity-induced aging process in terms of a mechanical stability landscape: as the active system ages, it randomly explores different regions of configuration space until it reaches an absorbing state that is sufficiently stable to resist melting. We expect our results to hold generally for active systems that can form a glassy phase, regardless of system size and topology. Our findings may be experimentally verified in e.g. dense suspensions of biological or artificial microswimmers confined to a liquid droplet interface or hydrogel.

## Methods

### Model system and dynamics simulations

Our active-matter system is composed of *N* interacting rods of length $\ell$ that all experience a constant self-propulsion force with magnitude *F* along their longitudinal rod axis $\hat{{\bf{u}}}$. In order to mimic steric repulsion between the particles, we represent each rod *i* as a rigid chain of *n* spherical segments ($n=\lceil 14\ell \mathrm{/8}\rceil$), and let every segment interact with all the segments of any other rod *j* through a repulsive Yukawa potential. The total interaction energy between a pair of rods is given by ${U}_{ij}=\frac{{U}_{0}}{{n}^{2}}{\sum }_{\alpha =1}^{n}\,{\sum }_{\beta =1}^{n}\,\frac{\exp (-{r}_{ij,\alpha \beta }/\lambda )}{{r}_{ij,\alpha \beta }}$, where *r*_*ij*,*αβ*_ is the Euclidean distance between segment *α* of rod *i* and segment *β* of rod *j*, *U*_0_ is the strength of the potential, and the screening length *λ* can be interpreted as the effective diameter of the segments. Note that in terms of computational costs, our force-calculation routine is effectively that of an (*N* × *n*)-particle system, rather than *N*.

We simulate the active-particle dynamics by integrating the overdamped Brownian equations of motion for the center-of-mass coordinates **r**_*i*_ and normalized orientation vector ${\hat{{\bf{u}}}}_{i}$ of each particle *i*. Explicitly, we consider the dynamics within the *local 2D plane tangential to the sphere* at position **r**_*i*_, project all segment coordinates and ${\hat{{\bf{u}}}}_{i}$ onto this plane, and solve

4 $$\begin{array}{rcl}{\dot{{\bf{r}}}}_{i} & = & {{\bf{D}}}_{T}[-{\nabla }_{{{\bf{r}}}_{i}}U+F{\hat{{\bf{u}}}}_{i}],\\ {\dot{\hat{{\bf{u}}}}}_{i} & = & -{{\bf{D}}}_{R}{\nabla }_{{\hat{{\bf{u}}}}_{i}}U,\end{array}$$

where the dots denote time derivatives, $U=\mathrm{(1/2)}\,{\sum }_{i,j\ne i}\,{U}_{ij}$, and ${\nabla }_{{\hat{{\bf{u}}}}_{i}}$ is the gradient on the unit circle. The matrices **D**_*T*_ and **D**_*R*_ represent inverse translational and rotational friction tensors, respectively, defined as

5 $${{\bf{D}}}_{T}={D}_{0}[{D}_{\parallel }{\hat{{\bf{u}}}}_{i}\otimes {\hat{{\bf{u}}}}_{i}+{D}_{\perp }({\bf{I}}-{\hat{{\bf{u}}}}_{i}\otimes {\hat{{\bf{u}}}}_{i})],$$

6 $${{\bf{D}}}_{R}={D}_{0}{D}_{R}{\bf{I}},$$

where *D*_0_ is the Stokesian diffusion coefficient, **I** is the 2 × 2 unit matrix, $\otimes$ is the dyadic product, and for the parameters ${D}_{\parallel }$, ${D}_{\perp }$, and *D*_*R*_ we use, as in refs 8 and 57, the standard expressions for rod-like macromolecules given in ref. 70,

7 $$\begin{array}{rcl}\quad \,\,2\pi {D}_{\parallel } & = & \mathrm{ln}(a)-0.207+0.980{a}^{-1}-0.133{a}^{-2},\\ \quad \,4\pi {D}_{\perp } & = & \mathrm{ln}(a)+0.839+0.185{a}^{-1}+0.233{a}^{-2},\\ \pi {a}^{2}{D}_{R}/3 & = & \mathrm{ln}(a)-0.662+0.917{a}^{-1}-0.050{a}^{-2}.\end{array}$$

After every time step in the propagation of Eq. (4), we project the coordinates and orientation vector ${\hat{{\bf{u}}}}_{i}$ onto the tangent plane at the particle’s new position **r**_*i*_. Finally, we note that the equations of motion (4) do not contain any stochastic terms, implying that the dynamics is fully deterministic and is governed solely by the repulsive pair interactions and self-propulsion forces.

Following ref. 57, we adopt characteristic units such that *λ* = 1, *F* = 1, and *D*_0_ = 1, implying that time is measured in units of *τ* = *λ*/(*D*_0_ *F*). We fix the strength of the interaction potential to *U*_0_ = 250 and include only segment-segment interactions that fall within a cutoff radius *r*_*c*_ = 6*λ*. For the remaining parameters in our simulations, namely the total particle number *N*, the rod aspect ratio *a*, and packing fraction *ϕ*, we typically use values of *N* = 400 or 800, 1.5 ≤ *a* ≤ 16, and 0.01 ≤ *ϕ* ≤ 0.7. All simulations are performed using an Euler integration scheme with a discrete time step of 0.01*τ*.

Independent starting configurations are produced by first placing all particles’ centers of mass randomly on a spherical surface with large radius ${R}_{0}\ge \sqrt{N\ell \lambda /\mathrm{(0.4}\pi )}$ (corresponding to dilute packing fractions *ϕ* ≤ 0.1), using spherical particle coordinates ${{\bf{r}}}_{i}\equiv (r,{\theta }_{i},{\phi }_{i})=({R}_{0},{\cos }^{-1}\,\mathrm{(2}{x}_{1}-1),2\pi {x}_{2})$, where *θ*_*i*_ and ${\phi }_{i}$ are the polar and azimuthal angles of particle *i*, respectively. The variables *x*_1_ and *x*_2_ are drawn randomly from a uniform distribution on the interval (0, 1) to ensure approximately uniform coverage on the spherical surface. Similarly, we generate random particle orientations on the unit sphere, ${\hat{{\bf{u}}}}_{i}=\mathrm{(1},{\cos }^{-1}\,(2{x}_{3}-1)\mathrm{,\; 2}\pi {x}_{4})$, where again *x*_3_ and *x*_4_ are random variates on (0, 1). We subsequently project these orientation vectors onto the local tangent plane at position **r**_*i*_ and normalize such that $|{\hat{{\bf{u}}}}_{i}|=1$. In order to remove any unphysical overlaps between rods, we randomly displace particles whose segment coordinates overlap to within a distance of *λ*. After generating such an overlap-free random configuration at very low density, we linearly decrease the sphere radius from *R*_0_ to the desired size *R* (corresponding to the desired packing fraction *ϕ*_init_) in 200 steps, thereby allowing the system to briefly equilibrate for a time duration of 1*τ* at every fixed radius. We then let the system equilibrate at *ϕ* = *ϕ*_init_ for a duration of 2000*τ*, and subsequently collect data for analysis over a period of 60000*τ*.

### Melting and revitrification dynamics protocol

A single breathing cycle starts at a packing fraction *ϕ*_init_, and is then diluted to *ϕ*_br_ < *ϕ*_init_ by linearly increasing the sphere radius *R* in 30 steps, allowing the system to briefly equilibrate at every new *R*-value for a duration of 10*τ*. The system is subsequently re-densified toward *ϕ*_init_ by linearly decreasing *R* again over 30 × 10*τ*, followed by a final stage in which we keep the packing fraction constant at *ϕ* = *ϕ*_init_ during 300*τ*. Note that the time it would take a single free rod of length $\ell =2\lambda$ to swim its own length is 13.32*τ*, and the total cycle period thus offers a reasonable compromise between a quasi-static and sudden quench.

The autocorrelation functions of the angular velocity are calculated based on the angular velocities in the final configuration of every full breathing cycle. For the passive (*F* = 0) reference case for *ϕ*_br_ = 0.38 [Fig. 4(a)], we find that all the instantaneous velocities **v**_*i*_ are virtually zero, thus obscuring the calculation of the angular velocities ${\hat{{\boldsymbol{\omega }}}}_{i}=({{\bf{r}}}_{i}\times {{\bf{v}}}_{i})/|{{\bf{r}}}_{i}||{{\bf{v}}}_{i}|$ with large numerical noise. In order to still probe any possible changes in the passive particle configuration, we have assumed ${{\bf{v}}}_{i}={\hat{{\bf{u}}}}_{i}$ in this case. As can be seen from the dashed lines in Fig. 4(a), we detect only very small displacements for passive particles (leading to a decorrelation of *C*(*t*, *t*_*w*_) from 1 to $\approx$0.97), and only at very short initial times (*t* < 5 cycles). Note that these marginal rearrangements are essentially a consequence of the softness of the pair interaction; if the particles would interact through a strictly hard potential, an overlap-free configuration would–in the absence of activity and noise–rigorously yield *C*(*t*, *t*_*w*_) = 1.

### Stability analysis

In order to quantify the stability of the particle configurations during aging, we use the total number of displaced particles *N*_*r*_ as a metric. More specifically, for a given aging trajectory, we first place every configuration that is formed after a full breathing cycle onto a new sphere of varying radius *R*_*s*_ (*R*_1_ > *R*_*s*_ > *R*_2_), where *R*_*s*_ is varied linearly in 500 steps from *R*_1_ to *R*_2_. We choose these upper and lower bounds of the sphere radius such that they correspond to packing fractions 0.1 < *ϕ* < 1.0. For every possible value of *R*_*s*_, we rescale all particle coordinates {**r**_*i*_} of the specific configuration such that |**r**_*i*_| = *R*_*s*_ and ensure that all rod orientations $\{{\hat{{\bf{u}}}}_{i}\}$ lie tangent to the sphere, and subsequently perform a dynamics simulation at fixed *R* = *R*_*s*_ for a total duration of 50*τ*. We then measure how many particles *N*_*r*_ have undergone a significant center-of-mass displacement Δ*r* during any time interval Δ*t* over the course of this simulation run. After some testing, we have found that a suitable stability criterion is Δ*r* = 0.13*λ* and Δ*t* = 10*τ*, which corresponds to a displacement of approximately 17% of a rod’s width during the time it would take a free rod with *a* = 2 to swim its own length ($\ell =2\lambda$). We designate a configuration at a certain *R*_*s*_ and corresponding packing fraction *ϕ* as stable if and only if *N*_*r*_ = 0, and denote the lowest and highest possible packing fractions with *N*_*r*_ = 0 as *ϕ*_min_ and *ϕ*_max_, respectively.

The dependence of the stability on the magnitude of the self-propulsion force, as shown in Fig. 6, was calculated by first performing a dynamics simulation of *N* = 800 particles with activity strength *F* = 1, undergoing 20 consecutive breathing cycles for *ϕ*_init_ = 0.5 and *ϕ*_br_ = 0.42. As above, we placed every particle configuration formed after a full breathing cycle onto a new sphere with varying radius *R*_1_ > *R*_*s*_ > *R*_2_ by rescaling all particle coordinates to |**r**_*i*_| = *R*_*s*_ and projecting all orientation vectors $\{{\hat{{\bf{u}}}}_{i}\}$ to the locally tangent plane. For every such set of initial particle coordinates, we equipped each particle with a constant self-propulsion strength 0 < *F* < 2.0 and subsequently simulated the dynamics for a time span of 50*τ*. We used the same stability criterion as above, Δ*r* = 0.13*λ* and Δ*t* = 10*τ*, and deem the system stable if *N*_*r*_ = 0. Note that one could also introduce a more refined stability criterion that is explicitly *F*-dependent; however, inspection by eye of the various trajectories for different *F*-values showed that our current criterion is reasonable for all cases considered. Furthermore, it may be seen from Fig. 6 that the resulting stability bounds *ϕ*_min_ and *ϕ*_max_ vary non-monotonously with *F*–an important point that would still hold for a monotonously changing choice of Δ*r*.

### Data availability

Data are available on request from the authors.

## Electronic supplementary material

Supplementary Information

Supplementary Movie S1

Supplementary Movie S2

Supplementary Movie S3

Supplementary Movie S4

Supplementary Movie S5

Supplementary Movie S6

Supplementary Movie S7

Supplementary Movie S8
