# Supplementary material for: Aging and rejuvenation of active matter under topological constraints
Source: Sci Rep. 2017 Jul 18;7:5667. doi: 10.1038/s41598-017-05569-6 (PMC5516002; doi:10.1038/s41598-017-05569-6)
Supplement: Supplementary file 1 — Supplementary Information [file 41598_2017_5569_MOESM1_ESM.pdf]

Supplementary Information for

"Aging and rejuvenation of active matter under topological constraints"

by Liesbeth M. C. Janssen, Andreas Kaiser, and Hartmut Löwen

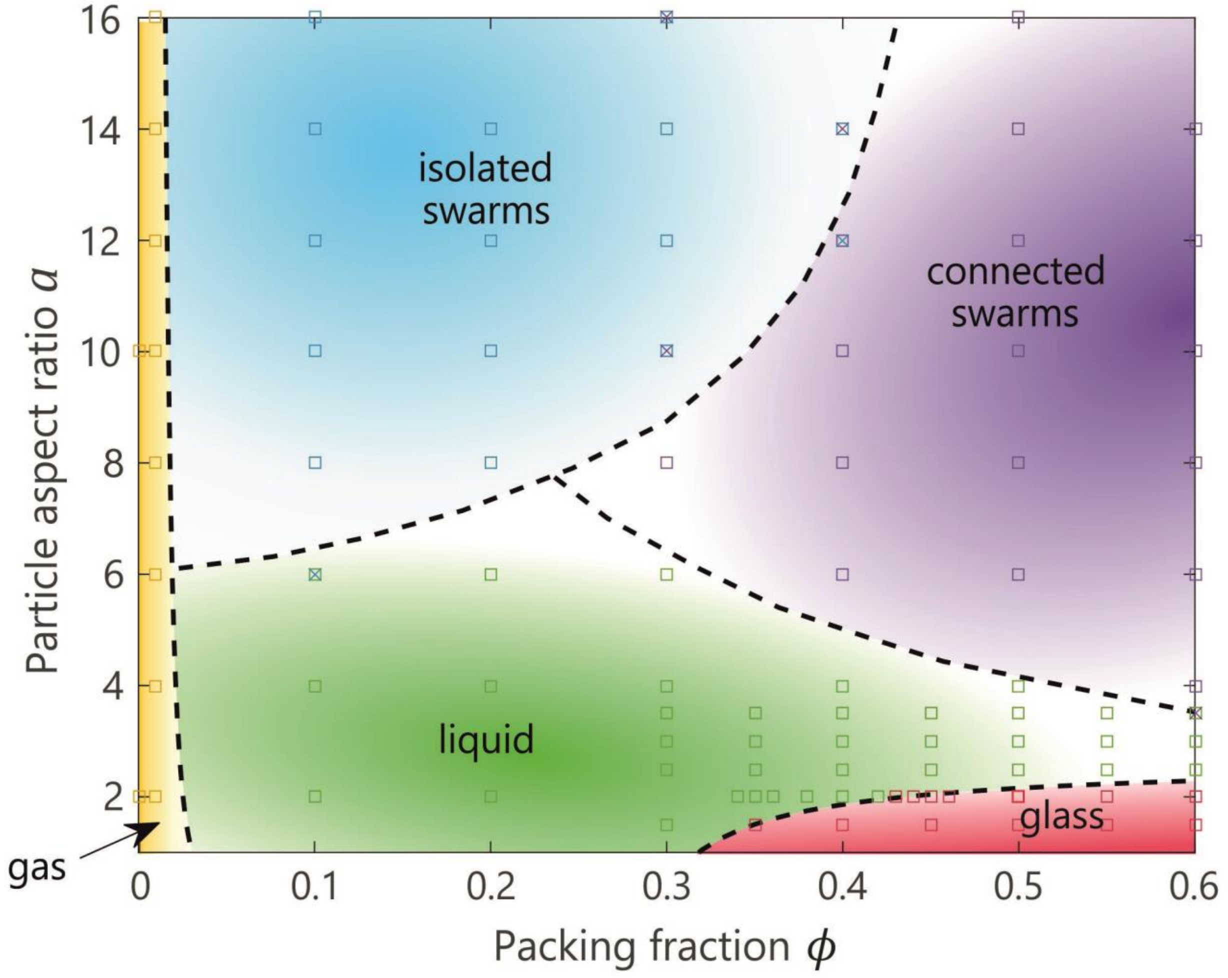

Figure S1: Non-equilibrium state diagram with evaluated state points indicated by squares.
